# Supplementary material for: Multi‐dataset identification of innovative feature genes and molecular mechanisms in keratoconus
Source: J Cell Mol Med. 2024 Sep 19;28(18):e70079. doi: 10.1111/jcmm.70079 (PMC11412914; doi:10.1111/jcmm.70079)
Supplement: Supplementary file 2 — Material S2. [file JCMM-28-e70079-s002.zip › 7.GSEA analysis/1.ARL11/KEGG_PATHWAYS_IN_CANCER.html]

Details for gene set KEGG\_PATHWAYS\_IN\_CANCER[GSEA]

|  || Dataset | ARL11.group.cls#h\_versus\_l |
| Phenotype | group.cls#h\_versus\_l |
| Upregulated in class | l |
| GeneSet | KEGG\_PATHWAYS\_IN\_CANCER |
| Enrichment Score (ES) | -0.24450265 |
| Normalized Enrichment Score (NES) | -1.0586041 |
| Nominal p-value | 0.37547892 |
| FDR q-value | 1.0 |
| FWER p-Value | 0.998 |
Table: GSEA Results Summary

  

Fig 1: Enrichment plot: KEGG\_PATHWAYS\_IN\_CANCER      
 Profile of the Running ES Score & Positions of GeneSet Members on the Rank Ordered List

  

| SYMBOL | TITLE | RANK IN GENE LIST | RANK METRIC SCORE | RUNNING ES | CORE ENRICHMENT || 1 | FGF16 | na | 91 | 0.669 | 0.0179 | No |
| 2 | RARB | na | 584 | 0.459 | 0.0177 | No |
| 3 | FGF14 | na | 927 | 0.398 | 0.0200 | No |
| 4 | TRAF5 | na | 1521 | 0.335 | 0.0130 | No |
| 5 | LAMB4 | na | 1566 | 0.332 | 0.0220 | No |
| 6 | APC2 | na | 1889 | 0.305 | 0.0220 | No |
| 7 | PIK3R3 | na | 2180 | 0.284 | 0.0222 | No |
| 8 | TGFBR2 | na | 2250 | 0.279 | 0.0288 | No |
| 9 | RET | na | 2305 | 0.276 | 0.0357 | No |
| 10 | FN1 | na | 2409 | 0.270 | 0.0410 | No |
| 11 | CSF1R | na | 2453 | 0.268 | 0.0480 | No |
| 12 | CCNE2 | na | 2488 | 0.266 | 0.0552 | No |
| 13 | PIK3CG | na | 2870 | 0.247 | 0.0517 | No |
| 14 | WNT5A | na | 3234 | 0.230 | 0.0482 | No |
| 15 | FGF23 | na | 3267 | 0.229 | 0.0543 | No |
| 16 | CBLB | na | 3964 | 0.202 | 0.0403 | No |
| 17 | PLCG1 | na | 4334 | 0.189 | 0.0353 | No |
| 18 | RAC3 | na | 4612 | 0.179 | 0.0328 | No |
| 19 | NTRK1 | na | 4617 | 0.179 | 0.0381 | No |
| 20 | WNT9A | na | 5395 | 0.155 | 0.0203 | No |
| 21 | DCC | na | 5487 | 0.152 | 0.0223 | No |
| 22 | BRCA2 | na | 5798 | 0.142 | 0.0177 | No |
| 23 | FZD4 | na | 5857 | 0.141 | 0.0203 | No |
| 24 | IGF1R | na | 5874 | 0.140 | 0.0242 | No |
| 25 | HGF | na | 5944 | 0.138 | 0.0264 | No |
| 26 | PDGFRA | na | 5971 | 0.138 | 0.0299 | No |
| 27 | PIK3R1 | na | 6079 | 0.135 | 0.0309 | No |
| 28 | PLCG2 | na | 6096 | 0.135 | 0.0346 | No |
| 29 | CASP8 | na | 6150 | 0.133 | 0.0372 | No |
| 30 | SOS1 | na | 6194 | 0.132 | 0.0400 | No |
| 31 | CCNA1 | na | 6558 | 0.123 | 0.0332 | No |
| 32 | IL6 | na | 6613 | 0.121 | 0.0353 | No |
| 33 | WNT10B | na | 6703 | 0.119 | 0.0364 | No |
| 34 | TRAF3 | na | 6905 | 0.114 | 0.0340 | No |
| 35 | AR | na | 6920 | 0.114 | 0.0371 | No |
| 36 | PDGFA | na | 7165 | 0.108 | 0.0333 | No |
| 37 | FLT3 | na | 7449 | 0.103 | 0.0283 | No |
| 38 | FGF9 | na | 7521 | 0.101 | 0.0293 | No |
| 39 | SMO | na | 7558 | 0.101 | 0.0314 | No |
| 40 | COL4A1 | na | 7590 | 0.100 | 0.0335 | No |
| 41 | TGFB2 | na | 7601 | 0.100 | 0.0363 | No |
| 42 | PTCH1 | na | 7694 | 0.098 | 0.0367 | No |
| 43 | CREBBP | na | 7701 | 0.098 | 0.0395 | No |
| 44 | FLT3LG | na | 7704 | 0.098 | 0.0424 | No |
| 45 | GLI3 | na | 7876 | 0.095 | 0.0404 | No |
| 46 | FGF7 | na | 7913 | 0.094 | 0.0422 | No |
| 47 | MECOM | na | 7964 | 0.093 | 0.0436 | No |
| 48 | FZD1 | na | 8051 | 0.091 | 0.0439 | No |
| 49 | SKP2 | na | 8159 | 0.089 | 0.0436 | No |
| 50 | LAMA5 | na | 8273 | 0.087 | 0.0430 | No |
| 51 | CDKN2A | na | 8387 | 0.085 | 0.0423 | No |
| 52 | SUFU | na | 8482 | 0.083 | 0.0421 | No |
| 53 | CTBP1 | na | 8519 | 0.082 | 0.0436 | No |
| 54 | WNT3A | na | 8640 | 0.080 | 0.0426 | No |
| 55 | IKBKB | na | 8698 | 0.079 | 0.0433 | No |
| 56 | PIK3R2 | na | 8783 | 0.078 | 0.0433 | No |
| 57 | FOS | na | 8788 | 0.078 | 0.0456 | No |
| 58 | MTOR | na | 8802 | 0.078 | 0.0476 | No |
| 59 | SMAD2 | na | 8821 | 0.078 | 0.0495 | No |
| 60 | VEGFC | na | 8842 | 0.077 | 0.0513 | No |
| 61 | FGF8 | na | 8904 | 0.076 | 0.0518 | No |
| 62 | FGF1 | na | 9240 | 0.071 | 0.0443 | No |
| 63 | AKT2 | na | 9348 | 0.070 | 0.0433 | No |
| 64 | TP53 | na | 9447 | 0.068 | 0.0426 | No |
| 65 | STAT5B | na | 9483 | 0.068 | 0.0436 | No |
| 66 | FGF11 | na | 9544 | 0.067 | 0.0439 | No |
| 67 | CASP3 | na | 9607 | 0.066 | 0.0441 | No |
| 68 | FGFR3 | na | 9769 | 0.063 | 0.0414 | No |
| 69 | RXRA | na | 9938 | 0.061 | 0.0384 | No |
| 70 | FGF12 | na | 9953 | 0.061 | 0.0398 | No |
| 71 | SOS2 | na | 9955 | 0.061 | 0.0417 | No |
| 72 | PIK3R5 | na | 10118 | 0.059 | 0.0388 | No |
| 73 | RALGDS | na | 10287 | 0.056 | 0.0356 | No |
| 74 | MYC | na | 10444 | 0.054 | 0.0328 | No |
| 75 | FADD | na | 10496 | 0.054 | 0.0329 | No |
| 76 | SMAD4 | na | 10504 | 0.054 | 0.0344 | No |
| 77 | APPL1 | na | 10611 | 0.052 | 0.0329 | No |
| 78 | PRKCA | na | 10617 | 0.052 | 0.0344 | No |
| 79 | BRAF | na | 10628 | 0.052 | 0.0357 | No |
| 80 | KRAS | na | 10630 | 0.052 | 0.0373 | No |
| 81 | MDM2 | na | 10671 | 0.052 | 0.0377 | No |
| 82 | FOXO1 | na | 10691 | 0.051 | 0.0387 | No |
| 83 | TCF7L2 | na | 10815 | 0.050 | 0.0366 | No |
| 84 | ARNT | na | 10906 | 0.048 | 0.0355 | No |
| 85 | CEBPA | na | 10959 | 0.048 | 0.0355 | No |
| 86 | BCR | na | 10999 | 0.048 | 0.0358 | No |
| 87 | MSH3 | na | 11101 | 0.046 | 0.0343 | No |
| 88 | EGFR | na | 11115 | 0.046 | 0.0353 | No |
| 89 | KITLG | na | 11152 | 0.046 | 0.0357 | No |
| 90 | HSP90AA1 | na | 11158 | 0.046 | 0.0370 | No |
| 91 | MAPK9 | na | 11167 | 0.046 | 0.0381 | No |
| 92 | VHL | na | 11342 | 0.043 | 0.0344 | No |
| 93 | FGFR2 | na | 11489 | 0.042 | 0.0314 | No |
| 94 | TRAF2 | na | 11495 | 0.042 | 0.0326 | No |
| 95 | FZD5 | na | 11914 | 0.037 | 0.0215 | No |
| 96 | LAMB2 | na | 11967 | 0.036 | 0.0212 | No |
| 97 | PGF | na | 12205 | 0.034 | 0.0153 | No |
| 98 | MAX | na | 12403 | 0.032 | 0.0106 | No |
| 99 | FZD6 | na | 12404 | 0.032 | 0.0116 | No |
| 100 | WNT8A | na | 12464 | 0.032 | 0.0108 | No |
| 101 | FZD3 | na | 12567 | 0.031 | 0.0088 | No |
| 102 | CBL | na | 12670 | 0.030 | 0.0068 | No |
| 103 | WNT1 | na | 12803 | 0.029 | 0.0038 | No |
| 104 | HDAC1 | na | 12805 | 0.029 | 0.0047 | No |
| 105 | CDKN2B | na | 13191 | 0.025 | -0.0057 | No |
| 106 | JUN | na | 13319 | 0.024 | -0.0087 | No |
| 107 | PIK3CA | na | 13512 | 0.023 | -0.0136 | No |
| 108 | RALBP1 | na | 13546 | 0.023 | -0.0138 | No |
| 109 | EGF | na | 13590 | 0.022 | -0.0144 | No |
| 110 | XIAP | na | 14006 | 0.019 | -0.0259 | No |
| 111 | VEGFB | na | 14052 | 0.018 | -0.0266 | No |
| 112 | SMAD3 | na | 14170 | 0.017 | -0.0295 | No |
| 113 | PTEN | na | 14202 | 0.017 | -0.0299 | No |
| 114 | RBX1 | na | 14278 | 0.017 | -0.0315 | No |
| 115 | STAT5A | na | 14398 | 0.016 | -0.0345 | No |
| 116 | BIRC2 | na | 14603 | 0.014 | -0.0400 | No |
| 117 | CTNNB1 | na | 14653 | 0.014 | -0.0410 | No |
| 118 | FGF13 | na | 14704 | 0.013 | -0.0420 | No |
| 119 | HSP90AB1 | na | 14766 | 0.013 | -0.0434 | No |
| 120 | CCNE1 | na | 14811 | 0.013 | -0.0443 | No |
| 121 | PLD1 | na | 14860 | 0.012 | -0.0453 | No |
| 122 | CTNNA1 | na | 15057 | 0.011 | -0.0507 | No |
| 123 | FGF10 | na | 15104 | 0.010 | -0.0517 | No |
| 124 | CHUK | na | 15127 | 0.010 | -0.0520 | No |
| 125 | MAPK10 | na | 15299 | 0.009 | -0.0567 | No |
| 126 | STAT1 | na | 15344 | 0.009 | -0.0577 | No |
| 127 | RASSF5 | na | 15399 | 0.008 | -0.0591 | No |
| 128 | GLI2 | na | 15544 | 0.007 | -0.0630 | No |
| 129 | FGF19 | na | 15552 | 0.007 | -0.0630 | No |
| 130 | PIAS1 | na | 15768 | 0.005 | -0.0691 | No |
| 131 | FASLG | na | 15958 | 0.004 | -0.0745 | No |
| 132 | EPAS1 | na | 16021 | 0.004 | -0.0762 | No |
| 133 | DAPK1 | na | 16033 | 0.004 | -0.0764 | No |
| 134 | ERBB2 | na | 16056 | 0.003 | -0.0769 | No |
| 135 | BIRC5 | na | 16081 | 0.003 | -0.0775 | No |
| 136 | KIT | na | 16102 | 0.003 | -0.0780 | No |
| 137 | WNT3 | na | 16162 | 0.003 | -0.0796 | No |
| 138 | RB1 | na | 16201 | 0.002 | -0.0807 | No |
| 139 | CCDC6 | na | 16222 | 0.002 | -0.0812 | No |
| 140 | RAC1 | na | 16390 | 0.001 | -0.0860 | No |
| 141 | MSH2 | na | 16407 | 0.001 | -0.0864 | No |
| 142 | E2F2 | na | 16509 | 0.000 | -0.0894 | No |
| 143 | CCND1 | na | 16546 | -0.000 | -0.0904 | No |
| 144 | LAMB1 | na | 16555 | -0.000 | -0.0906 | No |
| 145 | WNT10A | na | 16653 | -0.001 | -0.0934 | No |
| 146 | CDH1 | na | 16747 | -0.002 | -0.0961 | No |
| 147 | MAP2K2 | na | 16997 | -0.003 | -0.1032 | No |
| 148 | GSTP1 | na | 17007 | -0.003 | -0.1034 | No |
| 149 | CTBP2 | na | 17070 | -0.004 | -0.1051 | No |
| 150 | NCOA4 | na | 17320 | -0.006 | -0.1121 | No |
| 151 | HHIP | na | 17366 | -0.006 | -0.1133 | No |
| 152 | RALA | na | 17494 | -0.007 | -0.1167 | No |
| 153 | PRKCG | na | 17545 | -0.007 | -0.1180 | No |
| 154 | BAX | na | 17601 | -0.008 | -0.1193 | No |
| 155 | CYCS | na | 17719 | -0.008 | -0.1225 | No |
| 156 | E2F1 | na | 17721 | -0.008 | -0.1223 | No |
| 157 | RARA | na | 17917 | -0.010 | -0.1276 | No |
| 158 | AKT1 | na | 18085 | -0.011 | -0.1322 | No |
| 159 | TCF7 | na | 18185 | -0.012 | -0.1347 | No |
| 160 | PIAS3 | na | 18227 | -0.012 | -0.1355 | No |
| 161 | IGF1 | na | 18329 | -0.013 | -0.1381 | No |
| 162 | CDC42 | na | 18361 | -0.013 | -0.1386 | No |
| 163 | TPM3 | na | 18375 | -0.013 | -0.1385 | No |
| 164 | HRAS | na | 18435 | -0.013 | -0.1399 | No |
| 165 | FH | na | 18555 | -0.014 | -0.1429 | No |
| 166 | RALB | na | 18562 | -0.014 | -0.1426 | No |
| 167 | WNT4 | na | 18621 | -0.014 | -0.1439 | No |
| 168 | CDK4 | na | 18688 | -0.015 | -0.1453 | No |
| 169 | FGF3 | na | 18816 | -0.016 | -0.1485 | No |
| 170 | RAC2 | na | 18868 | -0.016 | -0.1495 | No |
| 171 | STK4 | na | 18997 | -0.017 | -0.1527 | No |
| 172 | JAK1 | na | 19011 | -0.017 | -0.1526 | No |
| 173 | JUP | na | 19046 | -0.017 | -0.1530 | No |
| 174 | BID | na | 19064 | -0.017 | -0.1530 | No |
| 175 | AXIN2 | na | 19144 | -0.018 | -0.1547 | No |
| 176 | ITGA2 | na | 19176 | -0.018 | -0.1551 | No |
| 177 | ITGA3 | na | 19278 | -0.019 | -0.1575 | No |
| 178 | BAD | na | 19342 | -0.019 | -0.1587 | No |
| 179 | DVL3 | na | 19519 | -0.021 | -0.1632 | No |
| 180 | STK36 | na | 19690 | -0.022 | -0.1674 | No |
| 181 | ABL1 | na | 19735 | -0.022 | -0.1680 | No |
| 182 | ITGA2B | na | 19828 | -0.023 | -0.1700 | No |
| 183 | DAPK2 | na | 19907 | -0.023 | -0.1716 | No |
| 184 | WNT5B | na | 19914 | -0.023 | -0.1710 | No |
| 185 | TGFBR1 | na | 19919 | -0.023 | -0.1704 | No |
| 186 | FZD10 | na | 20030 | -0.024 | -0.1729 | No |
| 187 | CBLC | na | 20116 | -0.024 | -0.1746 | No |
| 188 | RUNX1T1 | na | 20149 | -0.025 | -0.1748 | No |
| 189 | CDKN1B | na | 20317 | -0.026 | -0.1789 | No |
| 190 | CASP9 | na | 20358 | -0.026 | -0.1792 | No |
| 191 | GSK3B | na | 20728 | -0.029 | -0.1890 | No |
| 192 | LAMA4 | na | 20981 | -0.031 | -0.1954 | No |
| 193 | TRAF6 | na | 21028 | -0.032 | -0.1958 | No |
| 194 | MSH6 | na | 21102 | -0.032 | -0.1969 | No |
| 195 | DAPK3 | na | 21421 | -0.035 | -0.2051 | No |
| 196 | HSP90B1 | na | 21584 | -0.036 | -0.2087 | No |
| 197 | PML | na | 21643 | -0.036 | -0.2093 | No |
| 198 | HDAC2 | na | 21663 | -0.037 | -0.2087 | No |
| 199 | RHOA | na | 21886 | -0.038 | -0.2140 | No |
| 200 | BIRC3 | na | 21919 | -0.039 | -0.2137 | No |
| 201 | FGF22 | na | 22003 | -0.040 | -0.2149 | No |
| 202 | PIAS2 | na | 22018 | -0.040 | -0.2141 | No |
| 203 | ITGA6 | na | 22097 | -0.041 | -0.2151 | No |
| 204 | CUL2 | na | 22180 | -0.042 | -0.2162 | No |
| 205 | DVL2 | na | 22249 | -0.042 | -0.2169 | No |
| 206 | RXRB | na | 22268 | -0.043 | -0.2161 | No |
| 207 | PTK2 | na | 22303 | -0.043 | -0.2157 | No |
| 208 | FGF21 | na | 22495 | -0.045 | -0.2199 | No |
| 209 | WNT2 | na | 22576 | -0.046 | -0.2208 | No |
| 210 | MAPK3 | na | 22614 | -0.046 | -0.2205 | No |
| 211 | PAX8 | na | 22674 | -0.047 | -0.2208 | No |
| 212 | HIF1A | na | 22716 | -0.047 | -0.2205 | No |
| 213 | IKBKG | na | 22799 | -0.048 | -0.2214 | No |
| 214 | EGLN2 | na | 22816 | -0.048 | -0.2204 | No |
| 215 | MLH1 | na | 22834 | -0.048 | -0.2194 | No |
| 216 | GRB2 | na | 23043 | -0.051 | -0.2239 | No |
| 217 | FGF18 | na | 23239 | -0.053 | -0.2280 | No |
| 218 | ITGAV | na | 23305 | -0.053 | -0.2282 | No |
| 219 | TRAF4 | na | 23331 | -0.054 | -0.2273 | No |
| 220 | RAF1 | na | 23336 | -0.054 | -0.2257 | No |
| 221 | LAMC1 | na | 23462 | -0.055 | -0.2277 | No |
| 222 | TFG | na | 23494 | -0.056 | -0.2269 | No |
| 223 | PPARD | na | 23551 | -0.056 | -0.2268 | No |
| 224 | PIAS4 | na | 23627 | -0.058 | -0.2272 | No |
| 225 | PIK3CB | na | 23818 | -0.060 | -0.2309 | No |
| 226 | DVL1 | na | 23820 | -0.060 | -0.2290 | No |
| 227 | CKS1B | na | 23832 | -0.060 | -0.2275 | No |
| 228 | EP300 | na | 23862 | -0.060 | -0.2265 | No |
| 229 | RASSF1 | na | 23998 | -0.062 | -0.2285 | No |
| 230 | BCL2 | na | 24069 | -0.063 | -0.2286 | No |
| 231 | FAS | na | 24099 | -0.064 | -0.2275 | No |
| 232 | NFKBIA | na | 24454 | -0.069 | -0.2357 | No |
| 233 | TRAF1 | na | 24455 | -0.069 | -0.2336 | No |
| 234 | NOS2 | na | 24516 | -0.070 | -0.2332 | No |
| 235 | MAPK8 | na | 24525 | -0.070 | -0.2313 | No |
| 236 | TCF7L1 | na | 24530 | -0.070 | -0.2292 | No |
| 237 | MAPK1 | na | 24626 | -0.071 | -0.2298 | No |
| 238 | CDK6 | na | 24642 | -0.071 | -0.2281 | No |
| 239 | CSF3R | na | 24746 | -0.073 | -0.2288 | No |
| 240 | AXIN1 | na | 24756 | -0.073 | -0.2268 | No |
| 241 | E2F3 | na | 25055 | -0.077 | -0.2332 | No |
| 242 | SPI1 | na | 25187 | -0.078 | -0.2346 | No |
| 243 | STAT3 | na | 25255 | -0.079 | -0.2341 | No |
| 244 | TGFB1 | na | 25459 | -0.082 | -0.2374 | No |
| 245 | CRK | na | 25703 | -0.086 | -0.2419 | Yes |
| 246 | FZD9 | na | 25720 | -0.086 | -0.2397 | Yes |
| 247 | PIK3CD | na | 25759 | -0.087 | -0.2381 | Yes |
| 248 | COL4A2 | na | 25836 | -0.088 | -0.2377 | Yes |
| 249 | CDK2 | na | 25887 | -0.088 | -0.2364 | Yes |
| 250 | FGF20 | na | 25897 | -0.089 | -0.2339 | Yes |
| 251 | EGLN1 | na | 25924 | -0.089 | -0.2320 | Yes |
| 252 | SLC2A1 | na | 26160 | -0.093 | -0.2359 | Yes |
| 253 | ARAF | na | 26195 | -0.093 | -0.2341 | Yes |
| 254 | LAMC2 | na | 26469 | -0.098 | -0.2390 | Yes |
| 255 | PTGS2 | na | 26519 | -0.099 | -0.2374 | Yes |
| 256 | CTNNA2 | na | 26542 | -0.099 | -0.2350 | Yes |
| 257 | TPR | na | 26694 | -0.102 | -0.2362 | Yes |
| 258 | WNT7A | na | 26706 | -0.103 | -0.2334 | Yes |
| 259 | LAMB3 | na | 26727 | -0.103 | -0.2308 | Yes |
| 260 | LAMC3 | na | 26756 | -0.103 | -0.2285 | Yes |
| 261 | NFKB1 | na | 26767 | -0.104 | -0.2256 | Yes |
| 262 | COL4A4 | na | 26954 | -0.107 | -0.2277 | Yes |
| 263 | BMP4 | na | 27068 | -0.110 | -0.2276 | Yes |
| 264 | RUNX1 | na | 27212 | -0.113 | -0.2283 | Yes |
| 265 | TGFA | na | 27293 | -0.114 | -0.2271 | Yes |
| 266 | FZD8 | na | 27395 | -0.117 | -0.2265 | Yes |
| 267 | CDKN1A | na | 27494 | -0.119 | -0.2257 | Yes |
| 268 | AKT3 | na | 27547 | -0.120 | -0.2235 | Yes |
| 269 | MET | na | 27578 | -0.120 | -0.2207 | Yes |
| 270 | RAD51 | na | 27599 | -0.121 | -0.2175 | Yes |
| 271 | KLK3 | na | 27780 | -0.125 | -0.2189 | Yes |
| 272 | WNT7B | na | 27789 | -0.125 | -0.2153 | Yes |
| 273 | NRAS | na | 27790 | -0.126 | -0.2114 | Yes |
| 274 | CRKL | na | 27878 | -0.128 | -0.2100 | Yes |
| 275 | LAMA3 | na | 28015 | -0.131 | -0.2100 | Yes |
| 276 | WNT16 | na | 28062 | -0.132 | -0.2072 | Yes |
| 277 | ITGB1 | na | 28114 | -0.134 | -0.2046 | Yes |
| 278 | PTCH2 | na | 28260 | -0.138 | -0.2046 | Yes |
| 279 | NFKB2 | na | 28308 | -0.139 | -0.2017 | Yes |
| 280 | FZD2 | na | 28464 | -0.142 | -0.2018 | Yes |
| 281 | ARNT2 | na | 28475 | -0.143 | -0.1977 | Yes |
| 282 | COL4A6 | na | 28477 | -0.143 | -0.1934 | Yes |
| 283 | VEGFA | na | 28646 | -0.148 | -0.1937 | Yes |
| 284 | ZBTB16 | na | 28917 | -0.155 | -0.1968 | Yes |
| 285 | PPARG | na | 28947 | -0.155 | -0.1929 | Yes |
| 286 | BCL2L1 | na | 29160 | -0.161 | -0.1941 | Yes |
| 287 | RELA | na | 29186 | -0.162 | -0.1899 | Yes |
| 288 | CXCL8 | na | 29280 | -0.165 | -0.1875 | Yes |
| 289 | APC | na | 29306 | -0.166 | -0.1831 | Yes |
| 290 | FGF6 | na | 29398 | -0.169 | -0.1806 | Yes |
| 291 | PDGFRB | na | 29523 | -0.173 | -0.1789 | Yes |
| 292 | WNT6 | na | 29533 | -0.173 | -0.1738 | Yes |
| 293 | MMP2 | na | 30057 | -0.190 | -0.1832 | Yes |
| 294 | FZD7 | na | 30073 | -0.190 | -0.1778 | Yes |
| 295 | BMP2 | na | 30615 | -0.208 | -0.1871 | Yes |
| 296 | WNT9B | na | 30689 | -0.211 | -0.1828 | Yes |
| 297 | MAP2K1 | na | 31070 | -0.223 | -0.1870 | Yes |
| 298 | LAMA2 | na | 31294 | -0.231 | -0.1864 | Yes |
| 299 | FGF5 | na | 31584 | -0.243 | -0.1873 | Yes |
| 300 | WNT8B | na | 31805 | -0.253 | -0.1860 | Yes |
| 301 | LAMA1 | na | 31863 | -0.255 | -0.1798 | Yes |
| 302 | FGF17 | na | 32313 | -0.275 | -0.1844 | Yes |
| 303 | PDGFB | na | 32445 | -0.283 | -0.1795 | Yes |
| 304 | LEF1 | na | 32575 | -0.292 | -0.1743 | Yes |
| 305 | CTNNA3 | na | 32684 | -0.300 | -0.1682 | Yes |
| 306 | CSF2RA | na | 32761 | -0.305 | -0.1611 | Yes |
| 307 | RXRG | na | 33017 | -0.323 | -0.1585 | Yes |
| 308 | FGF2 | na | 33068 | -0.326 | -0.1500 | Yes |
| 309 | EGLN3 | na | 33106 | -0.330 | -0.1409 | Yes |
| 310 | TGFB3 | na | 33262 | -0.342 | -0.1349 | Yes |
| 311 | MMP1 | na | 33608 | -0.370 | -0.1336 | Yes |
| 312 | PRKCB | na | 33863 | -0.399 | -0.1287 | Yes |
| 313 | SHH | na | 33891 | -0.404 | -0.1171 | Yes |
| 314 | MMP9 | na | 33942 | -0.411 | -0.1060 | Yes |
| 315 | ETS1 | na | 33978 | -0.417 | -0.0942 | Yes |
| 316 | WNT11 | na | 33983 | -0.417 | -0.0815 | Yes |
| 317 | GLI1 | na | 34157 | -0.445 | -0.0729 | Yes |
| 318 | NKX3-1 | na | 34313 | -0.477 | -0.0627 | Yes |
| 319 | FGFR1 | na | 34437 | -0.510 | -0.0506 | Yes |
| 320 | MITF | na | 34468 | -0.521 | -0.0355 | Yes |
| 321 | FGF4 | na | 34644 | -0.638 | -0.0210 | Yes |
| 322 | WNT2B | na | 34700 | -0.762 | 0.0008 | Yes |
Table: GSEA details [plain text format]

  

Fig 2: KEGG\_PATHWAYS\_IN\_CANCER      
 Blue-Pink O' Gram in the Space of the Analyzed GeneSet

  

Fig 3: KEGG\_PATHWAYS\_IN\_CANCER: Random ES distribution      
 Gene set null distribution of ES for **KEGG\_PATHWAYS\_IN\_CANCER**
